# Combined effects of ambient particulate matter exposure and a high-fat diet on oxidative stress and steatohepatitis in mice

**DOI:** 10.1371/journal.pone.0214680

**Published:** 2019-03-28

**Authors:** Shibin Ding, Chunyan Yuan, Bingjie Si, Mengruo Wang, Shuyan Da, Lanxin Bai, Weidong Wu

**Affiliations:** 1 Department of nutrition and food hygiene, school of public health, Xinxiang Medical University, Xinxiang, Henan Province, PR China; 2 Henan Collaborative Innovation Center of Molecular Diagnosis and Laboratory Medicine, Xinxiang Medical University, Xinxiang, Henan Province, PR China; The Ohio State University, UNITED STATES

## Abstract

**Background:**

Chronic exposure to ambient particulate matter with aerodynamic diameters < 2.5 (PM_2.5_) induces oxidative injury and liver pathogenesis. The present study assessed the effect and mechanism of long-term, real-world airborne particulate matter (PM) exposure on oxidative stress and hepatic steatosis in the context of a standard chow diet (STD) and a high-fat diet (HFD); the study further explored whether a combination of PM exposure and HFD treatment exacerbates the adverse effects in mice.

**Methods:**

C57BL/6J mice fed with STD or HFD (41.26% kcal fat) were exposed to PM or filtered air (FA) for 5 months. Lipid metabolism, oxidative stress and liver pathogenesis were evaluated. Real-time PCR and western blotting were performed to determine gene expression and molecular signal transduction in liver.

**Results:**

Chronic airborne PM exposure impaired oxidative homeostasis, caused inflammation and induced hepatic steatosis in mice. Further investigation found that exposure to real-world PM increased the expression of hepatic Nrf2 and Nrf2-regulated antioxidant enzyme gene. The increased protein expression of the sterol regulatory element binding protein-1c (SREBP-1c) and fatty acid synthase (FAS) in the liver were also observed in PM-exposed groups. Furthermore, the combination of PM exposure and HFD treatment caused a synergistic effect on the changes of lipid accumulation oxidative stress, inflammation in the mouse liver.

**Conclusions:**

Through *in vivo* study, we reveal that the combination of real-world ambient PM exposure and HFD treatment aggravates hepatic lipid metabolism disorders, inflammation and oxidative stress. PM exposure may accelerate the progression to non-alcoholic steatohepatitis by regulating SREBP-1c/FAS regulatory axis.

## 1. Instruction

Nonalcoholic fatty liver disease (NAFLD) includes a spectrum of phenotypes ranging from hepatic steatosis (fatty infiltration in hepatocytes) to hepatic nonalcoholic steatohepatitis (NASH), NASH-related fibrosis and ultimately cirrhosis [1], which is considered the hepatic manifestation of metabolic syndrome. In Western countries, prevalence numbers for NAFLD have dramatically increased in the past decades and are estimated to up to 30% in the general population [2]. Recently, numerous epidemiological studies have indicated that exposure to particulate matter (PM) with an aerodynamic diameter less than 2.5 μm (PM_2.5_) is closely associated with an increased risk for metabolic syndrome [3–5].

According to the most widespread, accepted model of the “two hits” hypothesis for NAFLD [6, 7], hepatic steatosis represents the “first hit”, which is caused by the accumulation of excess triglycerides in hepatocytes due to abnormal hepatic lipid metabolism. The “first hit” increases the vulnerability of the liver to various “second hits” including increased oxidative stress, endoplasmic reticulum stress, pro-inflammatory cytokines, gut-derived bacterial endotoxins, insulin resistance. It is important to note that excess adiposity is related to increased oxidative stress and the production of pro-inflammatory cytokines in liver. As multihit model proposed, many “hit” factors may act in a cooperative manner to cause the development of NAFLD [8]. Recent studies suggested that PM_2.5_ exposure represents a significant “hit” that triggers a non-alcoholic steatohepatitis (NASH)-like phenotype, induces hepatic fibrosis and impairs hepatic glucose metabolism in murine models [9, 10]. Previous work by our group reported that long-term, ambient PM exposure induces hepatic fibrosis and increased ROS production in the liver in mice; the combination of PM exposure and a high-fat diet (HFD) aggravates hepatic fibrosis [11]. Under these above conditions, PM exposure elicited liver oxidative injury and presented synergistic effects with HFD on hepatic fibrosis, which is considered a significant “hit” for NAFLD and/or could evolve to NASH; NASH is characterized by hepatic steatosis, inflammation, and the development of oxidative stress [12, 13]. However, the molecular events that dictate the evolution of NASH induced by ambient PM exposure have not been thoroughly elucidated to date. Furthermore, whether the combination of PM exposure and HFD treatment will aggravate oxidative stress development and NASH remains unclear.

The transcription factor nuclear factor-erythroid 2-related factor 2 (Nrf2) is a basic leucine zipper transcription factor. Nrf2 regulates the transcriptional induction of antioxidant response elements, (AREs)-mediated antioxidant enzymes, electrophile-conjugating enzymes, ubiquitin/proteasomes, and chaperone and heat-shock proteins in response to the adverse effects of cellular oxidative stress, including ROS [14, 15]. In addition, exposure to ultrafine PM increased Nrf2-regulated stress responsive genes [16]. PM_2.5_ exposure for 12 weeks significantly increased the expressions of Nrf2 and Nrf2-regulated antioxidant genes, such as heme oxygenase 1 (HO-1), in C57BL/6J mice [17]. Therefore, the role of Nrf2 in activating antioxidant and detoxification genes against cellular oxidative stress in STD or HFD-fed mice during PM exposure still needs further investigation.

Our study aimed to investigate the effects of long-term, real-world ambient PM exposure on NASH in standard chow diet (STD) and HFD-fed mice (C57BL/6J mouse), as well as studying whether the combination of PM exposure and the HFD treatment exacerbates oxidative stress, inflammation and abnormal hepatic lipid metabolism. Since hepatic lipid metabolism disorders plays a key role in the progression of NASH, in our study, we investigated the impacts of ambient PM exposure on lipid metabolism in the liver, and we further explored the molecular mechanisms associated with abnormal hepatic lipid metabolism and NASH.

## 2. Materials and methods

### 2.1. Chemicals and reagents

The commercial kits for alanine aminotransferase (ALT), aspartate amino-transferase (AST), malondialdehyde (MDA), oxidized/reduced glutathione (GSSG/GSH), catalase (CAT) and glutathione (GSH) detection were obtained from the Nanjing Jiancheng Bioengineering Institute (Nanjing, China). Serum total triglycerides (TG) and total cholesterol (TC) kits were purchased from BIOSINO Biotechnology and Science, Inc. (Beijing, China). The TRIzol agent was purchased from Invitrogen, Inc. (Carlsbad, CA, USA). RNA reverse transcription reagents and the SYBR Premix Ex Taq II kits were obtained from TAKARA Bio, Inc. (Otsu, Shiga, Japan). Rabbit monoclonal F4/80 antibody and rabbit monoclonal anti-LC3B were purchased from Cell Signaling Technology (Billerica, MA, USA). Rabbit monoclonal β-actin antibody, rabbit polyclonal Nrf2, Lamin B1, peroxisome proliferator-activated receptor α (PPARα), peroxisome proliferator-activated receptor γ (PPARγ), SREBP-1c, FAS, acyl CoA oxidase 1 (ACOX1), stearoyl-CoA desaturase 1 (SCD1) antibodies, and the secondary antibody were obtained from Affinity Biosciences, Inc. (Cincinnati, OH, USA). All chemicals used were of the highest grade commercially available.

### 2.2. Animals

Experimental protocols and procedures described were approved by the Committee of the Ethics Animal Experiments at Xinxiang Medical University (Protocol Number: XXMU-2016-0007). The institutional guidelines for the care and use of laboratory animals were strictly carried out.

Male C57BL/6 wild-type mice (6 weeks) were purchased from Vital River Laboratory Animal Technology Co., Ltd. (Beijing, China). All mice were maintained under standard conditions with a 12-h light/dark cycle and with *ad libitum* access to standard rodent chow and distilled water. After a 7-day acclimatization period to the new environment, mice were randomly divided into four experimental groups (n = 10 per experimental group) and treated for 5 months. The STD-filtered air (FA) group and the HFD-FA group were exposed to FA and fed with an HFD and an STD, respectively; the STD-PM group and the HFD-PM group were exposed to ambient PM and fed with an HFD and an STD, respectively. The ingredient composition of HFD, and the calorie supply of the STD and HFD were described in our previous study [11]. Body weight was measured weekly.

### 2.3. Real-world ambient PM exposure protocol

Mice in the PM-exposure groups were subjected to real-world PM from October 2016 to February 2017 in Xinxiang (a city with serious air pollution in China) using a whole-body PM-exposure system (purchased from Beijing Huironghe Technology CO. LTD.) as we previously described [11]. Meanwhile, mice in the FA-exposure groups were subjected to an identical protocol with the exception of a high-efficiency particulate air filter to remove PM_2.5_ and PM_10_ from the air stream, as described previously [18]. The mice were exposed to either ambient PM or FA for 6 h/day, 7 days/week for 5 months. In order to avoid the effect of PM on animals in the housing environment, a high-efficiency filter (Minnesota Mining and Manufacturing Corporation, Minnesota, USA) was used to filter the air in the housing room. The concentrations in the two chambers and in the housing room were recorded daily.

### 2.4. Blood samples and tissue preparation

After 5 months of treatment, all mice were anesthetized with 20 mg/kg b.w. of pentobarbital. Blood samples were collected by cardiac puncture and then centrifuged (875 × g; 10 min; 4°C) to obtain the serum. Liver tissue samples were removed and weighed. A portion of the liver was fixed in 4% paraformaldehyde and embedded in paraffin for histological analyses; and the remainder was frozen in liquid nitrogen and stored at -80°C.

### 2.5. Determination of metabolic parameters

The enzyme-linked immunosorbent assay (ELISA) kits for measuring TNF-α and IL-6 in serum were from BioLegend (San Diego, CA, USA). The levels of hepatic MDA, reduced-glutathione (GSH), the GSSG/GSH ratio, the activity of CAT in the liver, and ALT and AST in the serum were determined using commercial assay kits (Nanjing Jiancheng Bioengineering Institute) according to the manufacturer’s protocols. Hepatic lipids were extracted as previously described [19], and then hepatic TG and TC levels were determined according to the manufacturer’s protocols (BIO-SINO Biotechnology and Science, Inc.).

### 2.6. Liver histomorphometry

Frozen liver samples were embedded in a Tissue-Tek OCT compound (Sakura FineTek USA, Torrance, CA, USA) and stained with Oil Red O (Sigma, St. Louis, USA) to evaluate fat accumulation. Liver tissues embedded in paraffin were cut into 5 μm-thick sections and stained with hematoxylin-eosin (H&E) for histological analysis. A point-counting method was used for assessing hepatic steatosis as previously described [20–22]. Briefly, 10 microscopic fields from each H&E section (five sections per animal) were selected at random for calculating the volume density (Vv) of liver steatosis. The pathologist was blinded to assess all the histopathological examinations.

### 2.7. Immunohistochemistry of F4/80 for the liver

To assess macrophage cell surface marker F4/80 in the liver, paraffin-embedded tissue sections (5 μm) of the liver were labeled with rabbit anti-F4/80 (diluted 1:250, Cell Signaling Technology) at 4°C overnight; next the slides were rinsed and incubated at room temperature for with the appropriate secondary antibody for 1 hour. For 5 specimens, F4/80-positive cells (F4/80^+^-cells) were quantified in 10 randomly selected microscopic fields relative to total cells.

### 2.8. Real-time PCR

Total RNA was isolated from the liver samples using the TRIzol agent (Invitrogen, Inc.) according to the supplier’s protocols. Next, purified RNA (1 μg) was used to generate first-strand cDNA with a Primescript RT Reagent Kit (Takara Bio) according to the manufacturer’s protocol. Real-time polymerase chain reaction (PCR) was performed using a qPCR SYBR Green Mix kit (Takara Bio) to analyze the mRNA expression levels of *Nrf2*, *HO-1*, *TNF-α*, *IL1β*, *IL-6*, *farnesoid X receptor(FXR)*, *PPARα*, *ACOX1*, *MCAD*, *CPT1*, *PPARγ*, *the sterol regulatory element binding protein-1C (SREBP-1c)*, *fatty acid synthase (FAS)* and *SCD1*. The primer sequences are listed in Table 1. Real-time quantitative PCR (qPCR) was performed on an ABI 7900HT machine (Applied Biosystems, Foster City, CA, USA). The relative expression level was calculated using the 2^-ΔΔCT^ method, and values were normalized to *β-actin* mRNA.

**Table 1:** Primers used for real-time PCR.

Gene	Forward primer (5`-3`)	Reverse primer (5`-3`)
*Nrf2*	CTGAACTCCTGGACGGGACTA	CGGTGGGTCTCCGTAAATGG
*HO-1*	GATAGAGCGCAACAAGCAGAA	CAGTGAGGCCCATACCAGAAG
*TNF-α*	CCCAGACCCTCACACTCAGATC	GCCACTCCAGCTGCTCCTC
*IL1β*	GAGGATACCACTCCCAACAGACC	AAGTGCATCATCGTTGTTCATACA
*IL-6*	CTGCAAGAGACTTCCATCCAGTT	AGGGAAGGCCGTGGTTGT
*FXR*	CCAACCTGGGTTTCTACCC	CACACAGCTCATCCCCTTT
*PPARα*	GGAGTGCAGCCTCAGCCAAGTT	AGGCCACAGAGCGCTAAGCTGT
*ACOX1*	GCCTTTGTTGTCCCTATCCGT	CTTCAGGTAGCCATTATCCATCTCT
*MCAD*	TAATCGGTGAAGGAGCAGGTTT	GGCATACTTCGTGGCTTCGT
*CPT1*	AAGAACATCGTGAGTGGCGTC	AGCACCTTCAGCGAGTAGCG
*PPARγ*	ACGCGGGCTGAGAAGTCACG	AGTTGGTGGGCCAGAATGGCA
*SREBP-1c*	GGCACTAAGTGCCCTCAACCT	TGCGCAGGAGATGCTATCTCCA
*FAS*	CCTGGATAGCATTCCGAACCT	AGCACATCTCGAAGGCTACACA
*SCD1*	GCTGAATAAATCTGCTGTCTTG	CCGTGCCTTGTAAGTTCTGTG
*β-actin*	TTCGTTGCCGGTCCACACCC	GCTTTGCACATGCCGGAGCC

Nrf2, nuclear factor E2-related factor 2; HO-1, heme oxygenase 1; FXR, farnesoid X receptor; PPARα, peroxisome proliferator-activated receptor α; ACOX1, acyl CoA oxidase 1; MCAD, medium-chain specific acyl-CoA dehydrogenase; CPT1, carnitine palmitoyltransferase I; PPARγ, peroxisome proliferator-activated receptor γ; SREBP-1c, the sterol regulatory element binding protein-1C; FAS, fatty acid synthase; SCD1, stearoyl-CoA desaturase 1.

### 2.9. Protein extraction and Western blotting

Nuclear proteins were extracted using a nuclear extraction kit for mice (Beyotime Institute of Biotechnology, Beijing, China). For extracting total protein, cells were lysed in RIPA extraction buffer (Beyotime Institute of Biotechnology, Beijing, China), liver tissues were homogenized with a RIPA extraction buffer, incubated on ice for 2 h, and the lysates were centrifuged at 12000 g for 10 minutes at 4°C. Sample protein concentrations were determined by a BCA assay (BOSTER, Wuhan, China). Equal quantities of proteins from liver tissues were loaded and separated by 10% and 12% sodium dodecyl sulfate polyacrylaminde gel electrophoresis (SDS-PAGE). The proteins were subsequently transferred to a PVDF membrane (0.45 mm, Bio-Rad). The membranes were blocked with 5% non-fat milk, washed for three cycles of ten minutes, incubated with primary antibodies at 4°C overnight, and then incubated with the appropriate secondary antibody for 1 h at room temperature. Protein bands on the membrane were detected with the Pierce ECL Western Blotting Substrate (Thermo Fisher Scientific, Inc., USA). Band density was quantified by ChemiDoc Quantity-One (Bio-Rad Laboratories) software. β-actin expression was used as the control reference. Lamin B1 was used as the nuclear protein control reference.

### 2.10. Statistical analysis

SPSS 13.0 was used for data analysis. The data were expressed as the mean ± standard deviation (mean ± SD). The main effects of the HFD treatment and PM exposure were analyzed a 2 × 2 factorial ANOVA. A 2 x 2 factorial ANOVA was also used to analyze the potential interaction of HFD treatment and PM exposure on metabolic and genetic parameters. These parameters included TG and TC, TNFα, IL-6 in serum and the liver, hepatic steatosis percentage, F4/80 cell/total cell ratio of the liver, the target protein expressions of SREBP-1c, FAS, SCD1, PPARγ, PPARα, ACOX1, LC3, nuclear Nrf2 in the liver, ALT and AST in serum, hepatic MDA, CAT, reduced GSH and GSSG/GSH ratio, the mRNA expressions of Nrf2, HO-1, IL-6, TNF-α and IL-1β in the liver. The mRNA expressions of SREBP-1c, FAS, FXR, SCD1, PPARγ, PPARα, ACOX1, MCAD, CPT1, as well as body weight and liver weight, were analyzed by a non-repeated ANOVA, and *post hoc* contrasts were examined by Bonferroni’s test. A *P*-value less than 0.05 was considered to be statistically significant.

## 3. Results

### 3.1. PM exposure concentration

**Fig 1:**
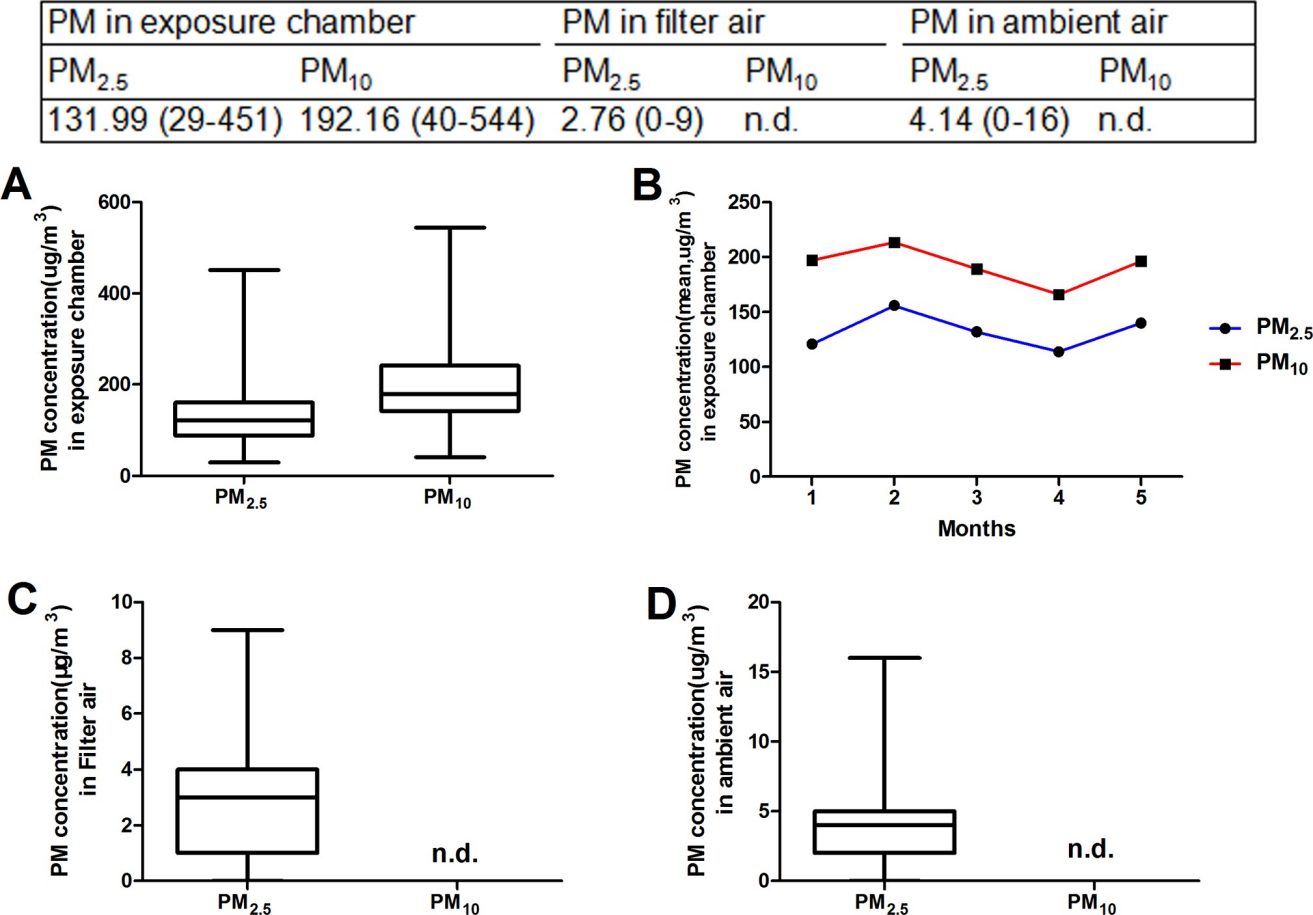
The concentration of PM_2.5_ and PM_10_ in different chambers (Median). (A) PM_2.5_ and PM_10_ in the exposure chamber. (B) The average concentration of PM in the exposure chamber. (C) PM_2.5_ and PM_10_ in the filter air. (D) PM_2.5_ and PM_10_ in the ambient air. n.d.: not detectable.

During exposure, the daily mean concentration of ambient PM_2.5_ and PM_10_ in the PM-exposure chamber were 131.99 μg/m^3^ and 192.16μg/m^3^, respectively (Fig 1A and 1B). Moreover, the daily mean concentration of ambient PM_2.5_ in the filtered-air and ambient housing room was 2.76 μg/m^3^ and 4.14 μg/m^3^, respectively (Fig 1C and 1D). The mean concentration of PM_2.5_ was an 8.8-fold concentration of the annual average PM_2.5_ National Ambient Air Quality Standard (15 μg/m^3^).

### 3.2. Body weight, liver weight, and lipid levels in the serum and liver

**Fig 2:**
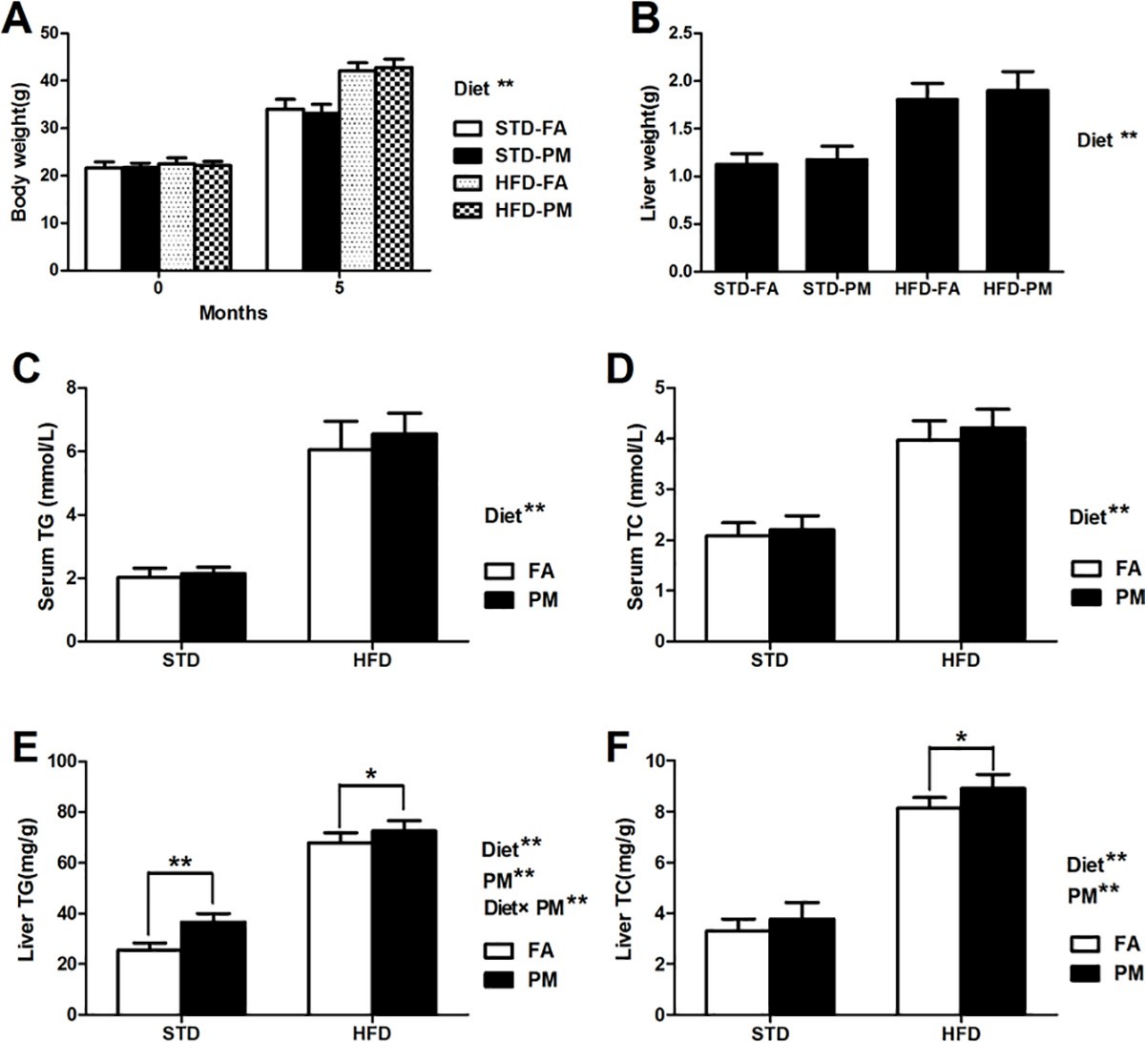
The body weight, liver weight, serum and hepatic lipid levels (n = 10). (A) Body weight. (B) Liver weight. (C) Serum TG levels. (D) Serum TC levels. (E) TG levels in liver. (F) TC levels in liver. Data are expressed as the mean ± SD. ^*^, *P*<0.05 and ^**^, *P*<0.01 compared with the STD-FA group. ^**##**^, *P*<0.01 compared with the STD-PM group. Diet^**^ indicates the HFD. PM^**^ indicates PM exposure. ^*^, *P*<0.05 and ^**^, *P*<0.01. Diet×PM indicates the interaction effect between HFD and PM exposure.

Changes in body weight and liver weight for the four groups were assessed (Fig 2A and 2B). There was no significant difference in body weight and liver weight between the STD-FA group and the STD-PM group (*P*>0.05), as well as between the HFD-FA group and the HFD-PM group (*P*>0.05). Moreover, compared to the STD-fed groups, body weight and liver weight significantly increased in the HFD-fed groups (*P*<0.01). The lipid in concentrations in the serum and liver were determined by commercial kits. There was no difference in the levels of TG (Fig 2C) or TC (Fig 2D) in serum between the PM-exposed groups and the control groups (*P*>0.05). However, TG and TC levels in the liver in the PM-exposed groups were significantly higher than in the control groups (*P*<0.01). In addition, TG (Fig 2E) and TC (Fig 2F) levels in the serum and liver in the HFD-fed groups were higher than those in the STD-fed groups (*P*<0.01). An interaction effect between the HFD treatment and PM exposure was observed for liver TG (*P*<0.01).

### 3.3. Ambient PM exposure leads to hepatic steatosis by impairing hepatic lipid metabolism

**Fig 3:**
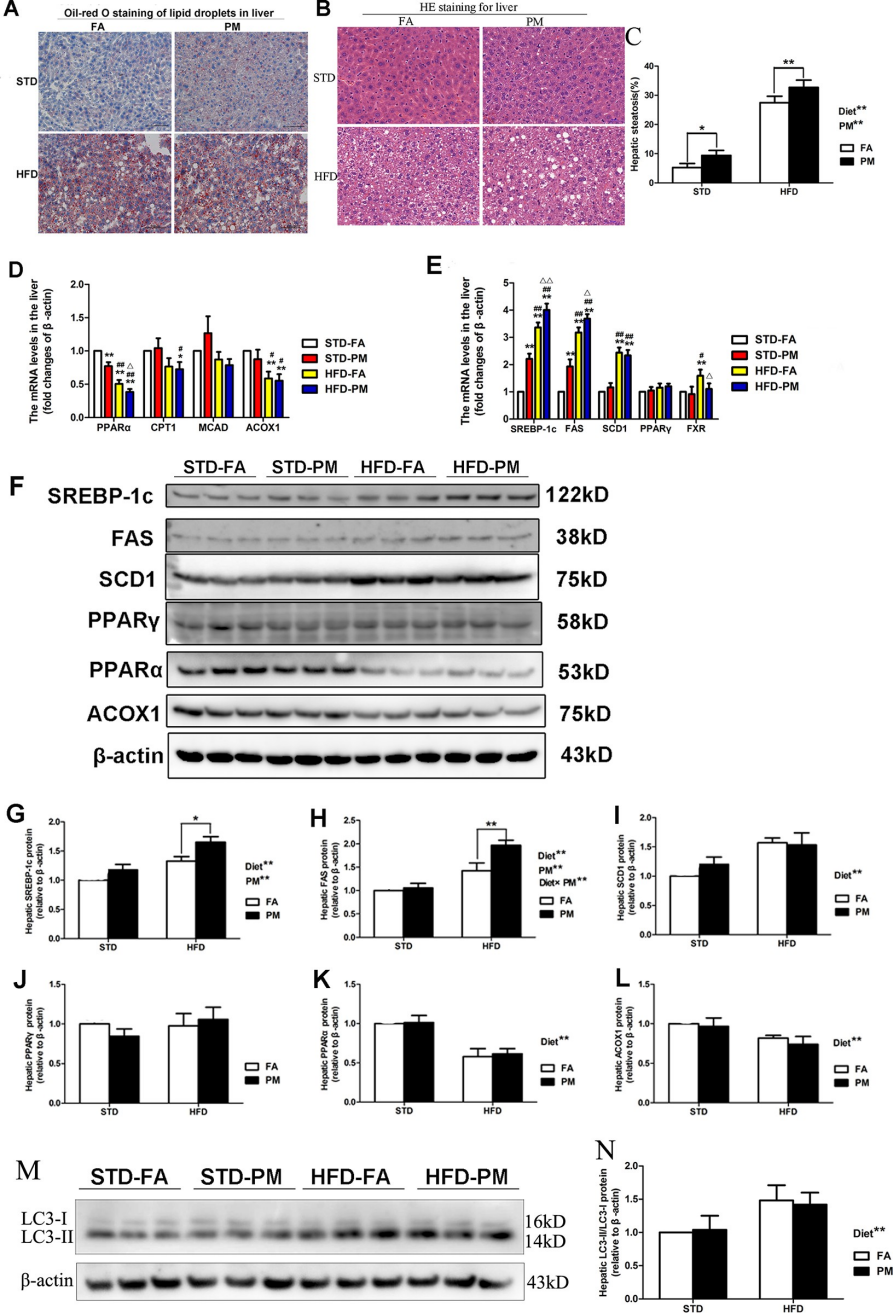
Ambient PM exposure leads to hepatic steatosis by impairing hepatic lipid metabolism. (A) Oil Red O staining observation of liver (×200, scale bars = 100 μm). (B) H&E staining observation of liver (×200, scale bars = 50 μm). (C) The volume density of quantitation of hepatic steatosis (n = 5). (D) The genes expression involved in fatty acid β-oxidation in liver (n = 5). (E) The mRNA expression of genes involved in lipogenesis and FXR in liver (n = 5). (F) Bands of PPARα, PPARγ, ACOX1, FAS, SREBP-1c, SCD1. (G) Protein expression of SREBP-1c (n = 3). (H) Protein expression of FAS (n = 3). (I) Protein expression of SCD1 (n = 3). (J) Protein expression of PPARγ (n = 3). (K) Protein expression of PPARα (n = 3). (L) Protein expression of ACOX1 (n = 3). (M) Bands of LC3-I and LC3-II. (N) The ratio of LC3-II and LC3-I (n = 3). Data are expressed as the mean ± SD. ^*^, *P*<0.05 and ^**^, *P*<0.01 compared with the STD-FA group. ^**#**^, *P*<0.05 and ^**##**^, *P*<0.01 compared with the STD-PM group. ^**Δ**^, *P*<0.05 and ^**ΔΔ**^, *P*<0.01 compared with the STD-PM group. Diet^**^ indicates the HFD. PM^**^ indicates PM exposure. ^*^, *P*<0.05 and ^**^, *P*<0.01. Diet×PM indicates the interaction effect between HFD and PM exposure.

The liver histological analyses (Oil Red O staining and H&E staining) of the four groups are presented in Fig 3A–3C. As indicated by the Oil Red O staining, accumulation of hepatic lipid droplets was increased in the liver of mice exposed to PM for 5 months (Fig 3A). Furthermore, mice subjected to HFD for 5 months showed a greater accumulation of lipid droplets compared to the STD-fed mice (Fig 3A). Consistent with hepatic lipid accumulation, the occurrence of hepatic steatosis was significantly higher in the mice exposed to PM than in the mice exposed to FA (*P*<0.01) (Fig 3B and 3C). Moreover, hepatic steatosis percentages were markedly higher in the HFD-treated mice than in the STD-fed mice (*P*<0.01) (Fig 3B and 3C). We checked the mRNA expression of some of the critical genes involved in triglyceride metabolism homeostasis using quantitative real-time PCR. As shown in Fig 3D, the mRNA expression of key genes involved in fatty acid β-oxidation, including PPARα, CPT1, MCAD and ACOX1, were determined. The mRNA expression of PPARα in the liver of STD-PM group was lower than that in the STD-FA group (*P*<0.01), and the mRNA expression of PPARα in liver of HFD-PM group was lower than the HFD-FA group (*P*<0.01). The mRNA expression of ACOX1 was significantly lower in the liver for the HFD-treated groups than in the liver for the STD-fed groups (*P*<0.01 or *P*<0.05). Compared to the STD-fed groups, the mRNA expression of CPT1 significantly decreased in the HFD-PM group (*P*<0.05). As shown in Fig 3E, the mRNA expression of SREBP-1c, FAS and SCD1, which participate in lipogenesis, significantly increased in the liver of the HFD-fed mice compared to the STD-fed mice (*P*<0.01). Additionally, compared with the mice in the FA-exposed group, the mRNA expression of SREBP-1c and FAS in the liver was significantly higher in the PM-exposed mice (*P*<0.01 or *P*<0.05). Furthetmore, the mRNA expression of FXR in the liver in the HFD-PM group was significantly lower than in the HFD-FA group (*P*<0.05). However, the mRNA expression of MCAD and PPARγin the liver was not significantly different among the four groups (*P*>0.05).

The immunoblotting analysis showed that the protein expression of SREBP-1c and FAS in the liver significantly increased in the PM-exposed mice (*P*<0.01) and the HFD-treated mice (*P*<0.01), compared to their control groups (Fig 3F–3H). Moreover, an interaction effect of PM exposure and the HFD treatment was observed for FAS protein expression (*P*<0.01) (Fig 3H). The protein expression of PPARα and ACOX1 significantly decreased, and the protein expression of SCD1 significantly increased in the liver of HFD-fed mice compared to STD-fed mice (Fig 3I–3K). Moreover, no significant difference in PPARγ protein expression was observed among the four groups (Fig 3J). In addition, we assessed the level of autophagy in liver by measuring microtubule-associated protein 1 A/1B-light chain 3 (LC3) protein expressions and LC3-II/LC3-I ratio in liver. Compared to STD-fed mice, the ratio of LC3-II vs LC3-I was significantly increased in the liver of HFD-fed mice (*P*<0.01) (Fig 3M and 3N). No significant difference of the ratio of LC3-II vs LC3-I was observed between the control groups and the PM-exposed groups (*P*>0.05) (Fig 3M and 3N).

### 3.4. Ambient PM exposure leads to liver injury and an oxidative response in the liver

**Fig 4:**
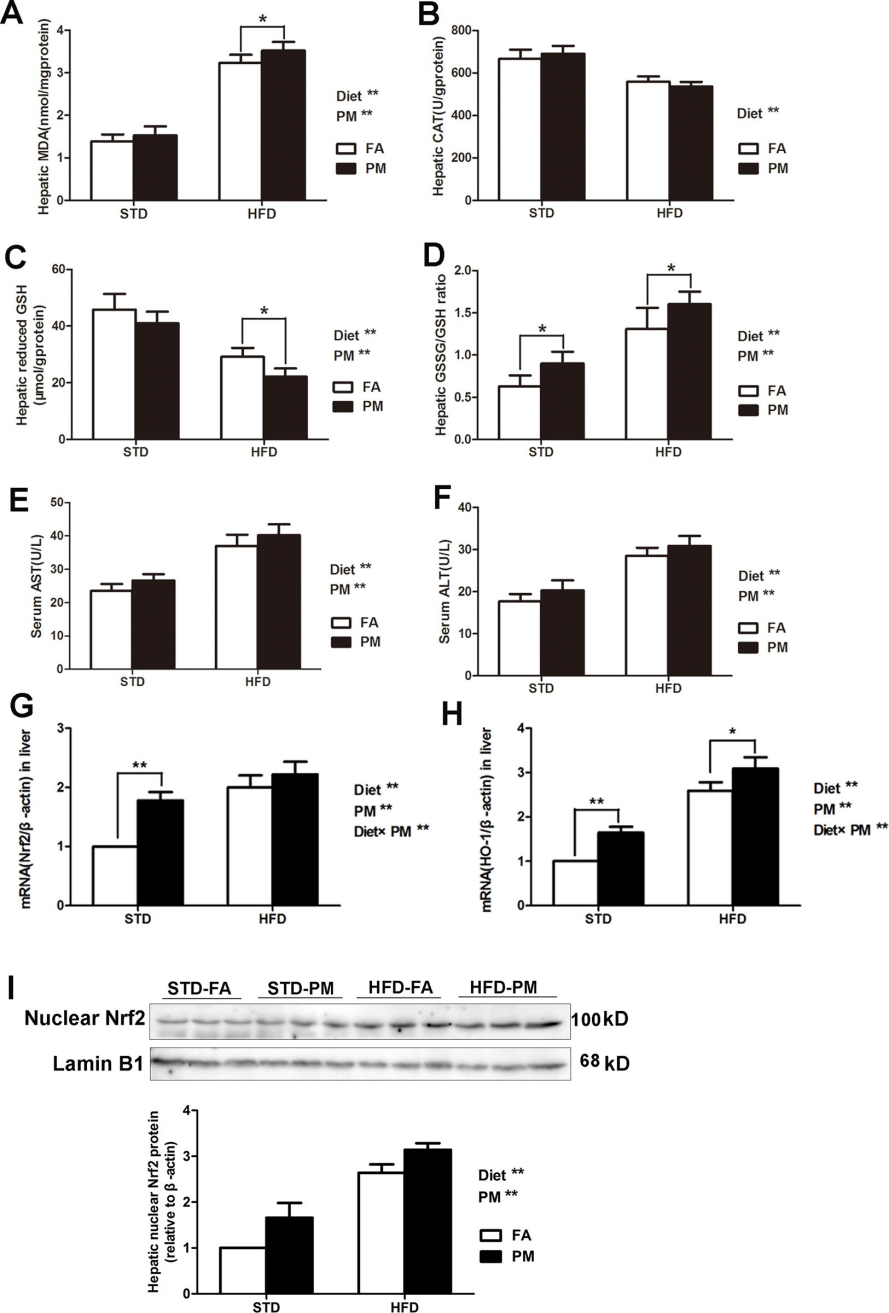
Ambient PM exposure induced liver injury and oxidative response in liver. (A) Hepatic MDA levels (n = 8–10). (B) Hepatic CAT levels (n = 8–10). (C) Hepatic reduced GSH levels (n = 8–10). (D) Hepatic GSSH/GSH ratio (n = 8–10). (E) Serum AST (n = 10). (F) Serum ALT (n = 10). (G) The mRNA expression of Nrf2 (n = 5). (H)The mRNA expression of HO-1(n = 5). (I) Immunoblotting of hepatic nuclear Nrf2 in four groups. (J) Immunoblotting analysis of hepatic nuclear Nrf2 in four groups (n = 3). Data are expressed as the mean ± SD. ^*^, *P*<0.05 and ^**^, *P*<0.01.Diet^**^ indicates the HFD. PM^**^ indicates PM exposure. Diet×PM indicates the interaction effect between HFD and PM exposure.

We assessed the activities of antioxidative enzymes in the liver of the four groups. As shown in Fig 4A–4D, a higher level of MDA and a higher GSSG/GSH ratio, as well as a lower level of reduced GSH, were observed in the livers of the HFD-treated group and the PM-exposed group compared to the control groups (*P*<0.01). Compared to the STD treatment, the HFD treatment significantly decreased the CAT levels in liver of the mice (*P*<0.01). No significant changes in the hepatic CAT level was observed between the PM-exposed group and the control group (*P*>0.05). As shown in Fig 4E and 4F, compared to the control group, significant increases in serum AST and ALT levels were observed in the HFD-fed groups and PM-exposed groups (*P*<0.01). As shown in Fig 4G and 4H, the oxidative response to PM exposure in the liver was measured in our study. Nrf2, a basic leucine zipper transcription factor, could regulate the transcriptional induction of antioxidant response element (ARE)-containing genes to encode antioxidant enzymes, such as HO-1, in response to ROS-induced cellular stress. The mRNA expression of Nrf2 and HO-1 was measured. Compared to the control group, the HFD treatment and PM exposure significantly increased the mRNA expression of Nrf2 and HO-1, as well as the protein expression of Nrf2 in the liver of the mice. An interaction effect between PM exposure and the HFD treatment was observed for the mRNA expression of Nrf2 and HO-1 in the liver (*P*<0.01). The nuclear protein expression of Nrf2 in the liver significantly increased in the HFD-fed groups and the PM-exposed groups compared to the control groups (*P*<0.01) (Fig 4I).

### 3.5. Pro-inflammatory cytokine levels in the serum and liver

**Fig 5:**
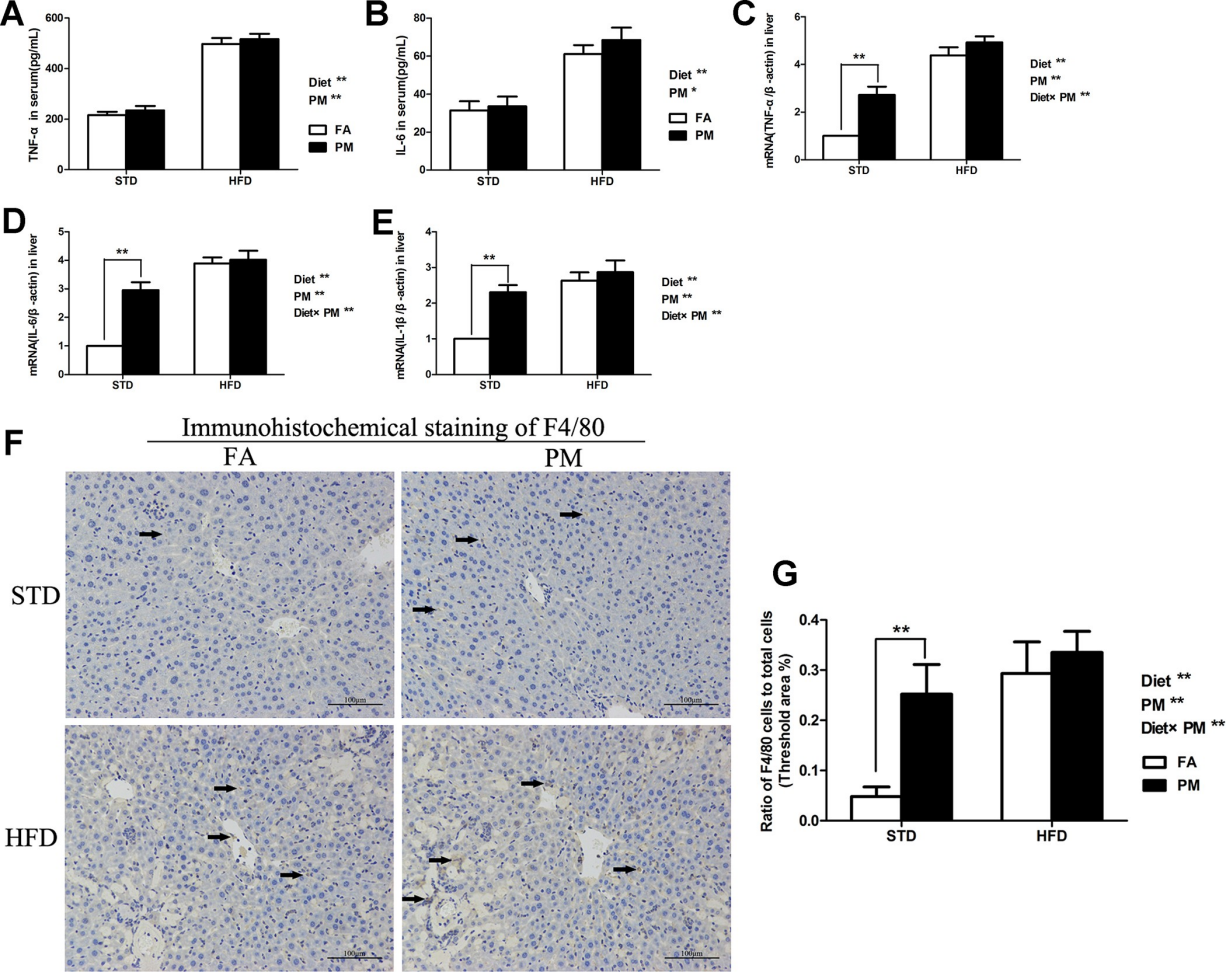
Ambient PM exposure activated inflammatory pathway via regulating JNK in liver. (A) The levels of TNF-α in serum (n = 8). (B) The levels of IL-6 in serum (n = 8). (C) The mRNA expression of TNF-α in liver (n = 5). (D) The mRNA expression of IL-6 in liver (n = 5). (E) The mRNA expression of IL-1β in liver (n = 5). (F) Immunohistochemical staining of macrophage cell surface marker F4/80 in liver (×200, scale bars = 100 μm). (G) The ratios of activated Kupffer cells (F4/80) to total cells (n = 5). Data are expressed as the mean ± SD. ^**^, *P*<0.01. Diet^**^ indicates the HFD. PM^**^ indicates PM exposure. Diet×PM indicates the interaction effect between HFD and PM exposure.

The inflammatory responses in HFD-fed and PM-exposed mice were assessed. The levels of TNF-α and IL-6 in serum determined by ELISA kits were significantly increased in the HFD-treated mice and PM-exposed mice compared to their control groups (*P*<0.01 or *P*<0.05) (Fig 5A and 5B). The mRNA expression of pro-inflammatory cytokines, including TNF-α, IL-6 and IL-1β, were significantly higher in the liver of the HFD-treated mice and PM-exposed mice compared to the control groups (*P*<0.01) (Fig 5C–5E). Furthermore, the hepatic mRNA levels of TNF-α, IL-6 and IL-1β were significantly higher in the STD-PM group than in the STD-FA group (*P*<0.01). No difference of TNF-α, IL-6 and IL-1β were observed between the HFD-PM group and the HFD group (*P*>0.05). A significant interaction effect between the HFD treatment and PM exposure was observed for the mRNA levels of TNF-α, IL-6 and IL-1β in the liver (*P*<0.01) (Fig 5C–5E).

### 3.6. Protein expression of macrophage cell surface makers, F4/80, in the liver

Immunohistochemical staining was used to assess the protein expression level of F4/80 in the liver. As shown in Fig 5F and 5G, the HFD treatment and PM exposure significantly increased the F4/80 level in the liver of the mice (*P*<0.01). In addition, the F4/80 level in the liver of the STD-PM group was significantly higher than in the STD-FA group (*P*<0.01) (Fig 5G). There was a significant interaction between PM exposure and the HFD treatment for F4/80 protein expression in the liver (*P*<0.01) (Fig 5G).

## 4. Discussion

In the present study, we demonstrated that exposure to environmentally relevant and real-world PM by a whole-body exposure system induces oxidative stress and NASH, characterized with hepatic inflammation, hepatic lipid metabolism disorder and steatosis, in mice. Furthermore, an interaction between PM exposure and the HFD treatment was observed for these adverse effects. An important finding in this study is that long-term PM exposure activated Nrf2-regulated oxidative stress in the liver, causing NASH by increasing lipogenic gene expression and triggering the JNK-mediated inflammation pathway in the liver. The combination of PM exposure and the HFD treatment enhanced these harmful effects.

Multiple factors including environmental factor, genetic susceptibility, gut microbiota and diet model are involved in the pathogenesis of NAFLD [23–26]. Previous studies suggested that ambient PM has the potential ability to induce NAFLD by different mechanisms, including induces the recruitment of Kupffer cell cytokine secretion in liver and causes a direct inflammation and oxidative damage to DNA in the liver [27]. Importantly, the effects of long-term exposure to ambient PM on specific molecular signal pathways of the hepatic lipid metabolism should be investigated.

The present study extends our previous observations on real-world PM exposure and its ability to increase hepatic ROS production causing liver fibrosis [11]. The harmful health effects of PM air pollution on the human body have been demonstrated to involve the mechanisms of oxidative stress and inflammation [28, 29]. Excess free fatty acids or lipid peroxidation could cause oxidative stress, which is a vital factor in the progression from steatosis to steatohepatitis [30]. Given the central role of oxidative stress in the development of NAFLD, we analyzed the expressions of Nrf2 (a crucial transcription factor that induces the expression of antioxidant enzymes and phase II detoxifying enzymes), Nrf2-regulated antioxidant gene (HO-1) and antioxidant enzymes (such as CAT activity, reduced-GSH level), as well as the GSSG/GSH ratio and MDA in the liver. Several studies have shown that the activating Nrf2 could reduce ROS production [31] and have a hepatoprotection effect in several models of NASH [32, 33]. After the PM challenge, Nrf2 was activated in lung and liver tissues [34] and Nrf2-regulated enzymes were induced in endothelial cells [35]. Our study showed that PM exposure activated Nrf2 signaling and increased Nrf2-regulated antioxidant gene HO-1 expression. In accordance with our study, Xu *et al*. has also reported that PM_2.5_ exposure increased the expressions of Nrf2 and Nrf2-regulated antioxidant gene (HO-1) in the liver [17]. Furthermore, PM exposure increased the hepatic MDA level, increased the GSSG/GSH ratio, and decreased the reduced-GSH level. Consumption of an HFD along with PM exposure can lead to oxidative stress and liver injury; therefore, the combination of the HFD treatment and PM exposure caused synergistic effects on Nrf2 and Nrf2-regulated antioxidant gene HO-1 expression in our study. These results suggest that the generation of ROS by PM exposure and the subsequent oxidative stress in the cell causes the accumulation of GSSG. It also depletes intracellular levels of reduced-GSH, leading to a high GSSG/GSH ratio and resulting in a “redox homeostasis” disorder.

Oxidative stress could activate c-JunN-terminal kinase (JNK) and nuclear factor-κB (NF-κB), leading to the induction of inflammatory genes. As previously reported, the JNK pathway plays a vital role in the pathogenesis of NAFLD [36]. Increased JNK activation contributed to hepatocyte death and liver injury induced by TNFα [37]. Exposure to PM_2.5_ for 10 weeks caused PM_2.5_ to be delivered to the liver and activated the Kupffer cells [38, 39], triggering inflammation pathways regulated by JNK, nuclear factor kappa B (NF-κB) and Toll-like receptor 4 (TLR4) in the liver [9]. It has also been reported that PM exposure could induce oxidative stress and inflammatory events in peripheral circulation [40]. In our study, long-term PM exposure increased the levels of pro-inflammatory cytokines (TNF-α and IL-6) in serum, causing inflammation-related cytokine gene expression in the liver tissue. We also found that the combination of the HFD treatment and PM exposure markedly exacerbated hepatic inflammation and macrophages infiltration in liver. These above results suggest that long-term exposure to real-world ambient PM could induce liver macrophage infiltration, which may contribute to systematic inflammation.

A previous study demonstrated that PM_2.5_ exposure for 10 weeks lead to a NASH-like phenotype characterized by steatosis, inflammation and fibrosis in the liver [9]. Additionally, exposure to PM_2.5_ for 4 weeks caused disruptions in hepatic glucose and lipid metabolism in KKAy mice (a genetically susceptible model of Type II diabetes mellitus) [41]. However, our mechanistic understanding behind the PM exposure-induced hepatic NASH-like phenotype is limited. In our study, we found that hepatic TG and TC markedly accumulated in PM-exposed mice; this finding is in agreement with results of another study, which reported that mice exposed to PM_2.5_ for 10 weeks accumulated TG and TC in the liver [9]. Consistent with previous research, we found increased hepatic intracellular ballooning degeneration and smaller lipid droplets. Based on the findings above, we further investigated the possible mechanism of PM in the excessive accumulation of lipids in the hepatocytes. Autophagy could regulate lipid metabolism by eliminating hepatocytes TG to prevent development of steatosis [42]. In this study, we found long-term PM_2.5_ exposure has no effect on hepatic autophagy in mice. In disagreement with our study, a previous study has reported that PM_2.5_ exposure for 10 weeks relieved hepatic steatosis in high-fat diet-induced obese mice through inducing hepatic autophagy in livers in a manner depending on the MyD88-mediated inflammatory pathway [43]. These different results indicate the effects of autophagy activation caused by environmental stress factor (such as PM exposure) may be overtaken by other hepatic lipid metabolic regulating effects during long-term ambient PM exposure. Hepatic lipid disorder mainly involves impaired fatty acid β-oxidation and upregulated *de novo* fat synthesis [44]. SREBP-1c, a key transcriptional factor, can regulate the expression of hepatic lipogenesis genes, such as FAS, SCD1 and ACC [45]. In many studies, increased expression of SREBP-1c has been observed in hepatic steatosis [46, 47]. A previous study reported that mice exposed to concentrated PM_2.5_ (mean concentration is 101.5 μg/m^3^) for 24 weeks accumulated TG and TC in serum and the liver, possibly by regulating genes involved in lipid metabolism [48]. In accordance with this study, our study found similar changes in genes involved in lipid metabolism. Furthermore, we found that PM exposure increased the protein expression of hepatic SREBP-1c, which further increased FAS protein expression in the liver. Intriguingly, hepatic dyslipidemia was aggravated by the HFD treatment in mice with the PM challenge. However, PM exposure had no effect on liver proteins involved in fatty acid β-oxidation, including PPARα, CPT1, ACOX1, MCAD, and PPARγ. Taken together, our results indicate that long-term PM exposure causes hepatic lipid accumulation via upregulating the SREBP-1c/FAS regulatory axis, and the combination of PM exposure and an HFD treatment aggravates lipid metabolism disorders.

## 5. Conclusions

In our present study, we revealed that long-term, real-world ambient PM exposure induced hepatic steatosis, and the classic lipogenesis-regulated axes (SREBP-1c/FAS) were responsible for the hepatic lipid accumulation in the PM-exposed mice. We also found that long-term PM exposure increased oxidative stress by activating the Nrf2 signaling pathway and caused inflammation which, in combination, may promote the development of NASH. Thus, PM exposure-induced oxidative stress and inflammation might be a risk factor for NASH, and high-fat consumption acts in synergy with PM to exacerbate NASH. Because of the mice model (HFD-fed) of NAFLD/NASH are similar but not equal to human NAFLD/NASH, the results observed in this study should be further proved in human study.

## Supporting information

S1 Table The data of our manuscript. (DOCX)

S1 Fig The protein levels of SREBP-1c in liver of four groups. (JPG)

S2 Fig The protein levels of FAS in liver of four groups. (JPG)

S3 Fig The protein levels of SCD1 in liver of four groups. (JPG)

S4 Fig The protein levels of PPARgamma in liver of four groups. (TIF)

S5 Fig The protein levels of PPARalpha in liver of four groups. (JPG)

S6 Fig The protein levels of Beta-Actin in liver of four groups. (TIF)

S7 Fig The protein levels of Nuclear Nrf2 in liver of four groups. (TIF)

S8 Fig The protein levels of LaminB1 in liver of four groups. (JPG)

S9 Fig The protein levels of LC3B in liver of four groups. (JPG)

S10 Fig The protein levels of Beta-Actin in liver of four groups. (JPG)

## Abbreviations

HFD: high-fat diet
FA: filtered air
PM: ambient particulate matter
PM_2.5_: PM with aerodynamic diameter less than 2.5 μm
NAFLD: Nonalcoholic fatty liver disease
NASH: non-alcoholic steatohepatitis
Nrf2: nuclear factor-erythroid 2-related factor 2
HO-1: heme oxygenase 1
AREs: antioxidant response elements
JNK: c-Jun N-terminal kinase
TNF-α: tumor necrosis factor-α
IL-6: Interleukin-6
IL-1β: Interleukin-1β
FXR: farnesoid X receptor
PPARα: peroxisome proliferator-activated receptor α
PPARγ: peroxisome proliferator-activated receptor γ
SREBP-1c: the sterol regulatory element binding protein-1c
FAS: fatty acid synthase
ACOX1: acyl CoA oxidase 1
SCD1: stearoyl-CoA desaturase 1
LC3: microtubule-associated protein 1 A/1B-light chain 3
MDA: malonaldehyde
GSSG/GSH: oxidized/reduced glutathione
CAT: catalase
GSH: glutathione
