# Supplementary material for: Combined effects of ambient particulate matter exposure and a high-fat diet on oxidative stress and steatohepatitis in mice
Source: PLoS One. 2019 Mar 28;14(3):e0214680. doi: 10.1371/journal.pone.0214680 (PMC6438678; doi:10.1371/journal.pone.0214680)
Supplement: S1 Table — (DOCX) [file pone.0214680.s001.docx]

**Table 1. The body weight (0-month) in four groups (g, n=10)**

| STD-FA | STD-PM | HFD-FA | HFD-PM |
| --- | --- | --- | --- |
| 23.6 | 22.7 | 21.1 | 20.9 |
| 19.8 | 21.8 | 22.4 | 22.5 |
| 20.8 | 20.6 | 20.4 | 22.3 |
| 21.7 | 21.5 | 22.4 | 22.4 |
| 20.6 | 20.3 | 23.3 | 23.8 |
| 20.3 | 20.9 | 22.3 | 22.6 |
| 22.6 | 21.9 | 20.6 | 21.2 |
| 22.6 | 22.8 | 24.1 | 21.3 |
| 22.7 | 21.7 | 23.8 | 21.2 |
| 21.9 | 22.9 | 23.8 | 22.7 |

**Table 2. The body weight(5-month) in four groups (g, n=10)**

| STD-FA | STD-PM | HFD-FA | HFD-PM |
| --- | --- | --- | --- |
| 36.3 | 34.1 | 40.3 | 44.3 |
| 33.9 | 30.9 | 42.8 | 39.1 |
| 32.8 | 32.9 | 41.7 | 42.3 |
| 30.3 | 34.8 | 43.4 | 44.6 |
| 32.1 | 32.1 | 41.0 | 42.6 |
| 32.6 | 32.9 | 41.5 | 43.3 |
| 35.9 | 37.2 | 42.5 | 44.9 |
| 35.6 | 33.1 | 45.9 | 43.5 |
| 36.6 | 31.6 | 41.2 | 40.6 |
| 33.5 | 32.3 | 40.3 | 42.3 |

**Table 3. Liver weight(g)**

| STD-FA | STD-PM | HFD-FA | HFD-PM |
| --- | --- | --- | --- |
| 1.04 | 1.13 | 1.73 | 1.86 |
| 1.31 | 1.26 | 2.18 | 1.72 |
| 1.09 | 1.37 | 1.83 | 2.17 |
| 1.13 | 1.35 | 1.73 | 1.93 |
| 0.91 | 1.09 | 1.89 | 1.79 |
| 1.27 | 0.94 | 1.67 | 1.69 |
| 1.15 | 1.05 | 1.53 | 2.03 |
| 1.09 | 1.24 | 1.83 | 1.92 |
| 1.17 | 1.27 | 1.89 | 1.66 |
| 1.10 | 1.06 | 1.78 | 2.24 |

**Table 4. Serum TG in four groups (mmol/L, n=10)**

| STD-FA | STD-PM | HFD-FA | HFD-PM |
| --- | --- | --- | --- |
| 2.23 | 2.31 | 5.24 | 5.61 |
| 2.39 | 2.14 | 5.63 | 6.89 |
| 1.98 | 2.19 | 7.12 | 5.58 |
| 2.19 | 1.72 | 7.21 | 6.64 |
| 2.26 | 2.21 | 7.1 | 7.31 |
| 2.29 | 2.44 | 6.75 | 6.33 |
| 1.58 | 2.11 | 5.04 | 7.48 |
| 1.73 | 1.87 | 5.23 | 6.83 |
| 2.04 | 2.17 | 5.94 | 6.15 |
| 1.63 | 2.25 | 5.21 | 6.81 |

**Table 5. Serum TC in four groups (mmol/L, n=10)**

| STD-FA | STD-PM | HFD-FA | HFD-PM |
| --- | --- | --- | --- |
| 1.69 | 2.11 | 3.37 | 3.83 |
| 2.17 | 2.59 | 4.17 | 4.21 |
| 2.15 | 2.31 | 3.98 | 4.22 |
| 1.9 | 1.61 | 4.49 | 4.36 |
| 2.38 | 2.28 | 3.78 | 4.15 |
| 1.75 | 2.44 | 3.6 | 3.35 |
| 2.21 | 2.03 | 4.29 | 4.47 |
| 2.19 | 2.24 | 3.91 | 4.56 |
| 2.47 | 1.97 | 4.49 | 4.41 |
| 1.9 | 2.38 | 3.67 | 4.56 |

**Table 6. Hepatic TG in four groups (mg/g, n=10)**

| STD-FA | STD-PM | HFD-FA | HFD-PM |
| --- | --- | --- | --- |
| 23.68 | 35.58 | 73.12 | 64.85 |
| 21.21 | 32.19 | 69.44 | 73.96 |
| 30.17 | 41.12 | 72.38 | 70.12 |
| 24.72 | 37.85 | 70.85 | 75.6 |
| 27.35 | 33.56 | 67.87 | 76.43 |
| 25.12 | 38.44 | 60.56 | 68.45 |
| 24.88 | 36.69 | 65.98 | 74.2 |
| 23.81 | 33.08 | 68.21 | 70.06 |
| 29.45 | 42.11 | 62.83 | 73.85 |
| 25.39 | 35.23 | 67.31 | 77.93 |

**Table 7. Hepatic TC in four groups (mg/g, n=10)**

| STD-FA | STD-PM | HFD-FA | HFD-PM |
| --- | --- | --- | --- |
| 3.23 | 4.13 | 8.56 | 9.12 |
| 3.51 | 4.96 | 8.12 | 9.85 |
| 2.78 | 3.24 | 8.21 | 7.91 |
| 3.71 | 3.6 | 8.45 | 8.72 |
| 2.94 | 4.59 | 7.73 | 8.45 |
| 4.07 | 4.01 | 7.7 | 8.7 |
| 3.7 | 2.93 | 8.76 | 9.28 |
| 3.06 | 3.35 | 7.45 | 9.45 |
| 3.45 | 3.66 | 8.14 | 9.08 |
| 2.59 | 3.21 | 8.27 | 8.58 |

**Table 8. Hepatic steatosis(%, n=5)**

| STD-FA | STD-PM | HFD-FA | HFD-PM |
| --- | --- | --- | --- |
| 3.04 | 7.12 | 28.96 | 31.84 |
| 4.77 | 8.31 | 24.68 | 34.78 |
| 6.12 | 10.51 | 30.19 | 29.55 |
| 5.85 | 9.57 | 27.52 | 35.71 |
| 6.52 | 11.44 | 26.03 | 31.59 |

**Table 9. The F4/80 of liver in four groups (%, n=5)**

| STD-FA | STD-PM | HFD-FA | HFD-PM |
| --- | --- | --- | --- |
| 0.023 | 0.321 | 0.328 | 0.341 |
| 0.048 | 0.186 | 0.219 | 0.388 |
| 0.073 | 0.223 | 0.334 | 0.307 |
| 0.059 | 0.224 | 0.232 | 0.281 |
| 0.037 | 0.307 | 0.354 | 0.358 |

**Table 10. The serum TNFα in four groups (pg/mL, n=8)**

| STD-FA | STD-PM | HFD-FA | HFD-PM |
| --- | --- | --- | --- |
| 229.6 | 217.9 | 523.6 | 484.1 |
| 207.3 | 252.5 | 465.3 | 546.7 |
| 209.1 | 228.2 | 492.6 | 523.4 |
| 212.8 | 242.7 | 482.1 | 531.1 |
| 226.2 | 255.5 | 529.0 | 505.9 |
| 235.5 | 208.8 | 495.2 | 513.7 |
| 194.7 | 248.2 | 518.5 | 533.6 |
| 210.4 | 225.6 | 476.8 | 498.8 |

**Table 11. The serum IL-6 in four groups (pg/mL, n=8)**

| STD-FA | STD-PM | HFD-FA | HFD-PM |
| --- | --- | --- | --- |
| 36.5 | 28.5 | 57.8 | 68.9 |
| 34.8 | 39.5 | 63.4 | 65.5 |
| 29.3 | 35.6 | 61.2 | 71.8 |
| 25.5 | 27.8 | 68.4 | 79.7 |
| 31.9 | 41.4 | 60.1 | 63.6 |
| 30.4 | 32.3 | 66.4 | 62.1 |
| 24.7 | 34.5 | 54.7 | 74.9 |
| 38.1 | 29.2 | 57.1 | 62.2 |

**Table 12. The serum ALT in four groups (n=10)**

| STD-FA | STD-PM | HFD-FA | HFD-PM |
| --- | --- | --- | --- |
| 16.91 | 21.95 | 25.72 | 29.84 |
| 21.03 | 19.42 | 29.78 | 33.17 |
| 17.44 | 20.95 | 28.59 | 32.77 |
| 15.08 | 19.72 | 30.07 | 34.71 |
| 16.87 | 17.27 | 26.13 | 28.17 |
| 17.88 | 22.69 | 29.81 | 31.94 |
| 17.48 | 23.87 | 27.77 | 30.65 |
| 19.38 | 21.86 | 31.94 | 29.74 |
| 18.98 | 17.82 | 27.37 | 30.44 |
| 15.86 | 17.07 | 27.57 | 26.95 |

**Table 13. The serum AST in four groups (n=10)**

| STD-FA | STD-PM | HFD-FA | HFD-PM |
| --- | --- | --- | --- |
| 27.03 | 27.85 | 38.53 | 41.84 |
| 23.73 | 23.29 | 37.19 | 38.25 |
| 24.3 | 24.57 | 32.88 | 44.34 |
| 21.21 | 25.8 | 36.67 | 42.98 |
| 22.59 | 28.67 | 33.11 | 36.63 |
| 25.98 | 25.64 | 43.55 | 40.65 |
| 21.86 | 26.39 | 37.55 | 45.17 |
| 25.93 | 27.88 | 33.41 | 37.43 |
| 19.76 | 29.37 | 39.54 | 38.85 |
| 23.34 | 26.69 | 37.66 | 35.86 |

**Table 14. The hepatic CAT of liver (n=8)**

| STD-FA | STD-PM | HFD-FA | HFD-PM |
| --- | --- | --- | --- |
| 713.7 | 687.3 | 553.3 | 512.7 |
| 621.2 | 713.9 | 589.8 | 552.0 |
| 658.6 | 667.4 | 521.4 | 537.1 |
| 643.2 | 688.1 | 589.7 | 566.4 |
| 669.5 | 646.2 | 539.9 | 522.0 |
| 608.1 | 725.7 | 558.0 | 509.1 |
| 722.3 | 745.8 | 576.6 | 561.2 |
| 700.4 | 640.6 | 538.8 | 523.8 |

**Table 15. The hepatic GSH of liver (n=8)**

| STD-FA | STD-PM | HFD-FA | HFD-PM |
| --- | --- | --- | --- |
| 47.9 | 37.55 | 29.8 | 25.48 |
| 40.11 | 38.92 | 26.34 | 21.35 |
| 36.51 | 43.88 | 24.56 | 24.67 |
| 49.21 | 40.34 | 28.71 | 18.51 |
| 52.38 | 35.6 | 32.67 | 17.65 |
| 45.19 | 39.19 | 30.88 | 22.4 |
| 51.34 | 44.46 | 33.52 | 24.89 |
| 43.56 | 47.87 | 26.9 | 22.37 |

**Table 16. The hepatic GSSH/GSH of liver (n=8)**

| STD-FA | STD-PM | HFD-FA | HFD-PM |
| --- | --- | --- | --- |
| 0.75 | 0.79 | 1.59 | 1.43 |
| 0.57 | 0.88 | 1.23 | 1.59 |
| 0.43 | 1.02 | 1.07 | 1.48 |
| 0.82 | 1.17 | 0.93 | 1.71 |
| 0.66 | 0.89 | 1.21 | 1.66 |
| 0.71 | 0.91 | 1.34 | 1.47 |
| 0.48 | 0.86 | 1.49 | 1.89 |
| 0.6 | 0.7 | 1.64 | 1.58 |

**Table 17. The hepatic MDA of liver (n=8)**

| STD-FA | STD-PM | HFD-FA | HFD-PM |
| --- | --- | --- | --- |
| 1.45 | 1.79 | 3.18 | 3.38 |
| 1.18 | 1.85 | 3.49 | 3.84 |
| 1.58 | 1.48 | 2.93 | 3.66 |
| 1.27 | 1.39 | 3.12 | 3.2 |
| 1.33 | 1.66 | 3.44 | 3.5 |
| 1.51 | 1.4 | 3.27 | 3.42 |
| 1.2 | 1.37 | 3.08 | 3.56 |
| 1.56 | 1.29 | 3.34 | 3.63 |

**Table 18. The SREBP1c protein expression (relative to STD-FA group) of liver in four groups (n=3)**

| STD-FA | STD-PM | HFD-FA | HFD-PM |
| --- | --- | --- | --- |
| 1 | 1.28 | 1.27 | 1.55 |
| 1 | 1.16 | 1.30 | 1.74 |
| 1 | 1.10 | 1.41 | 1.68 |

**Table 19. The FAS protein expression (relative to STD-FA group) of liver in four groups (n=3)**

| STD-FA | STD-PM | HFD-FA | HFD-PM |
| --- | --- | --- | --- |
| 1 | 1.09 | 1.27 | 1.85 |
| 1 | 0.95 | 1.39 | 2.07 |
| 1 | 1.13 | 1.60 | 1.98 |

**Table 20. The SCD1 protein expression (relative to STD-FA group) of liver in four groups (n=3)**

| STD-FA | STD-PM | HFD-FA | HFD-PM |
| --- | --- | --- | --- |
| 1 | 1.06 | 1.66 | 1.31 |
| 1 | 1.25 | 1.55 | 1.58 |
| 1 | 1.30 | 1.49 | 1.72 |

**Table 21. The PPARγ protein expression (relative to STD-FA group) of liver in four groups (n=3)**

| STD-FA | STD-PM | HFD-FA | HFD-PM |
| --- | --- | --- | --- |
| 1 | 0.94 | 1.14 | 1.20 |
| 1 | 0.76 | 0.84 | 1.07 |
| 1 | 0.83 | 0.95 | 0.90 |

**Table 22. The PPARα** **protein expression (relative to STD-FA group) of liver in four groups (n=3)**

| STD-FA | STD-PM | HFD-FA | HFD-PM |
| --- | --- | --- | --- |
| 1 | 1.04 | 0.67 | 0.68 |
| 1 | 1.09 | 0.60 | 0.63 |
| 1 | 0.92 | 0.47 | 0.55 |

**Table 23. The ACOX1** **protein expression (relative to STD-FA group) of liver in four groups (n=3)**

| STD-FA | STD-PM | HFD-FA | HFD-PM |
| --- | --- | --- | --- |
| 1 | 0.93 | 0.79 | 0.63 |
| 1 | 0.89 | 0.85 | 0.81 |
| 1 | 1.09 | 0.82 | 0.78 |

**Table 24. The Nuclear Nrf2** **protein expression (relative to STD-FA group) of liver in four groups (n=3)**

| STD-FA | STD-PM | HFD-FA | HFD-PM |
| --- | --- | --- | --- |
| 1 | 1.42 | 2.67 | 2.98 |
| 1 | 1.53 | 2.80 | 3.19 |
| 1 | 2.03 | 2.45 | 3.26 |

**Table 25. The mRNA expressions of TNFα** **(relative to STD-FA group) (n=5)**

| STD-FA | STD-PM | HFD-FA | HFD-PM |
| --- | --- | --- | --- |
| 1.0 | 2.35 | 3.91 | 4.86 |
| 1.0 | 3.23 | 4.43 | 5.03 |
| 1.0 | 2.87 | 4.18 | 4.68 |
| 1.0 | 2.45 | 4.62 | 4.75 |
| 1.0 | 2.69 | 4.74 | 5.31 |

**Table 26. The mRNA expressions of IL-6** **(relative to STD-FA group) (n=5)**

| STD-FA | STD-PM | HFD-FA | HFD-PM |
| --- | --- | --- | --- |
| 1.0 | 3.26 | 3.94 | 4.27 |
| 1.0 | 2.78 | 4.11 | 3.73 |
| 1.0 | 2.59 | 3.78 | 4.32 |
| 1.0 | 3.19 | 4.02 | 4.12 |
| 1.0 | 2.94 | 3.59 | 3.66 |

**Table 27. The mRNA expressions of** **IL-1β (relative to STD-FA group) (n=5)**

| STD-FA | STD-PM | HFD-FA | HFD-PM |
| --- | --- | --- | --- |
| 1.0 | 2.06 | 2.78 | 2.87 |
| 1.0 | 2.32 | 2.34 | 2.96 |
| 1.0 | 2.41 | 2.88 | 2.61 |
| 1.0 | 2.19 | 2.43 | 2.72 |
| 1.0 | 2.56 | 2.71 | 3.21 |

**Table 28. The mRNA expressions of** **Nrf2 (relative to STD-FA group) (n=5)**

| STD-FA | STD-PM | HFD-FA | HFD-PM |
| --- | --- | --- | --- |
| 1.0 | 1.96 | 1.95 | 2.16 |
| 1.0 | 1.75 | 2.13 | 1.91 |
| 1.0 | 1.61 | 1.78 | 2.45 |
| 1.0 | 1.88 | 1.89 | 2.26 |
| 1.0 | 1.7 | 2.27 | 2.33 |

**Table 29. The mRNA expressions of** **HO-1 (relative to STD-FA group) (n=5)**

| STD-FA | STD-PM | HFD-FA | HFD-PM |
| --- | --- | --- | --- |
| 1.0 | 1.59 | 2.76 | 2.76 |
| 1.0 | 1.71 | 2.48 | 3.08 |
| 1.0 | 1.48 | 2.81 | 3.26 |
| 1.0 | 1.62 | 2.54 | 2.94 |
| 1.0 | 1.83 | 2.37 | 3.42 |

**Table 30. The mRNA expressions of** **PPARα** **(relative to STD-FA group) (n=5)**

| STD-FA | STD-PM | HFD-FA | HFD-PM |
| --- | --- | --- | --- |
| 1.0 | 0.85 | 0.59 | 0.36 |
| 1.0 | 0.69 | 0.47 | 0.38 |
| 1.0 | 0.77 | 0.5 | 0.33 |
| 1.0 | 0.8 | 0.44 | 0.45 |
| 1.0 | 0.75 | 0.53 | 0.4 |

**Table 31. The mRNA expressions of** **ACOX1** **(relative to STD-FA group) (n=5)**

| STD-FA | STD-PM | HFD-FA | HFD-PM |
| --- | --- | --- | --- |
| 1.0 | 0.89 | 0.66 | 0.45 |
| 1.0 | 1.07 | 0.51 | 0.69 |
| 1.0 | 0.78 | 0.72 | 0.52 |
| 1.0 | 0.94 | 0.48 | 0.5 |
| 1.0 | 0.7 | 0.57 | 0.61 |

**Table 32. The mRNA expressions of PPARγ (relative to STD-FA group) (n=5)**

| STD-FA | STD-PM | HFD-FA | HFD-PM |
| --- | --- | --- | --- |
| 1.0 | 0.89 | 1.23 | 1.14 |
| 1.0 | 1.12 | 1.26 | 1.26 |
| 1.0 | 0.95 | 1.03 | 1.17 |
| 1.0 | 1.08 | 0.95 | 1.09 |
| 1.0 | 1.21 | 1.3 | 1.34 |

**Table 33. The mRNA expressions of CPT1 (relative to STD-FA group) (n=5)**

| STD-FA | STD-PM | HFD-FA | HFD-PM |
| --- | --- | --- | --- |
| 1.0 | 0.94 | 0.85 | 0.76 |
| 1.0 | 1.23 | 0.67 | 0.87 |
| 1.0 | 0.85 | 0.78 | 0.58 |
| 1.0 | 1.08 | 0.61 | 0.69 |
| 1.0 | 1.11 | 0.92 | 0.73 |

**Table 34. The mRNA expressions of MCAD (relative to STD-FA group) (n=5)**

| STD-FA | STD-PM | HFD-FA | HFD-PM |
| --- | --- | --- | --- |
| 1.0 | 1.68 | 0.86 | 0.77 |
| 1.0 | 1.31 | 1.02 | 0.85 |
| 1.0 | 1.02 | 0.79 | 0.68 |
| 1.0 | 1.2 | 0.74 | 0.91 |
| 1.0 | 1.12 | 0.95 | 0.73 |

**Table 35. The mRNA expressions of FAS (relative to STD-FA group) (n=5)**

| STD-FA | STD-PM | HFD-FA | HFD-PM |
| --- | --- | --- | --- |
| 1.0 | 1.79 | 3.13 | 3.50 |
| 1.0 | 2.23 | 3.43 | 3.94 |
| 1.0 | 1.58 | 2.95 | 3.59 |
| 1.0 | 2.11 | 3.08 | 3.67 |
| 1.0 | 1.95 | 3.29 | 3.73 |

**Table 36. The mRNA expressions of SCD1 (relative to STD-FA group) (n=5)**

| STD-FA | STD-PM | HFD-FA | HFD-PM |
| --- | --- | --- | --- |
| 1.0 | 1.26 | 2.46 | 2.58 |
| 1.0 | 1.38 | 2.71 | 2.31 |
| 1.0 | 1.07 | 2.38 | 2.49 |
| 1.0 | 0.97 | 2.19 | 2.21 |
| 1.0 | 1.13 | 2.45 | 2.08 |

**Table 37. The mRNA expressions of FXR (relative to STD-FA group) (n=5)**

| STD-FA | STD-PM | HFD-FA | HFD-PM |
| --- | --- | --- | --- |
| 1.0 | 1.36 | 1.712 | 1.01 |
| 1.0 | 1.12 | 1.46 | 1.26 |
| 1.0 | 1.19 | 1.62 | 1.39 |
| 1.0 | 0.84 | 1.49 | 1.11 |
| 1.0 | 0.90 | 1.76 | 1.28 |

**Table 38. The mRNA expressions of SREBP1c (relative to STD-FA group) (n=5)**

| STD-FA | STD-PM | HFD-FA | HFD-PM |
| --- | --- | --- | --- |
| 1.0 | 2.23 | 3.32 | 4.12 |
| 1.0 | 2.08 | 3.65 | 4.29 |
| 1.0 | 2.45 | 3.18 | 4.07 |
| 1.0 | 1.97 | 3.41 | 3.85 |
| 1.0 | 2.31 | 3.26 | 3.68 |
