# Supplementary figures and images for: Combined effects of ambient particulate matter exposure and a high-fat diet on oxidative stress and steatohepatitis in mice
Source: PLoS One. 2019 Mar 28;14(3):e0214680. doi: 10.1371/journal.pone.0214680 (PMC6438678; doi:10.1371/journal.pone.0214680)

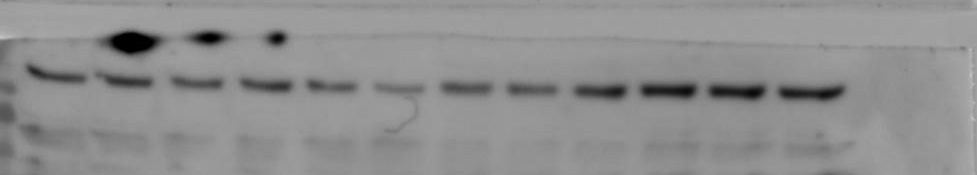

Supplement: S1 Fig — (JPG) [file pone.0214680.s002.jpg]

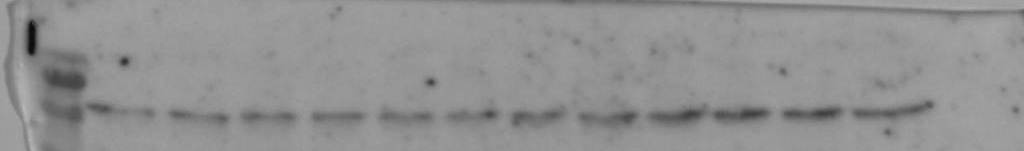

Supplement: S2 Fig — (JPG) [file pone.0214680.s003.jpg]

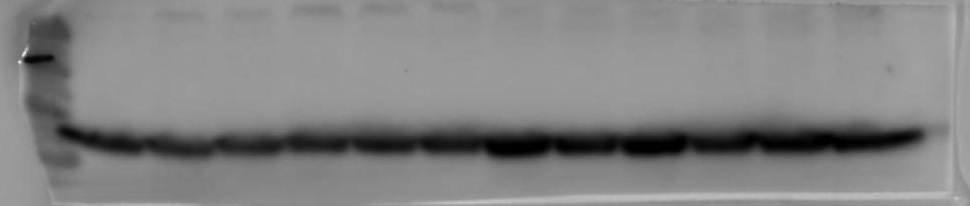

Supplement: S3 Fig — (JPG) [file pone.0214680.s004.jpg]

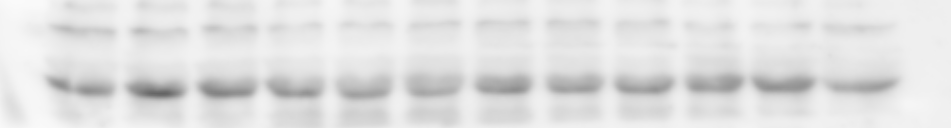

Supplement: S4 Fig — (TIF) [file pone.0214680.s005.tif]

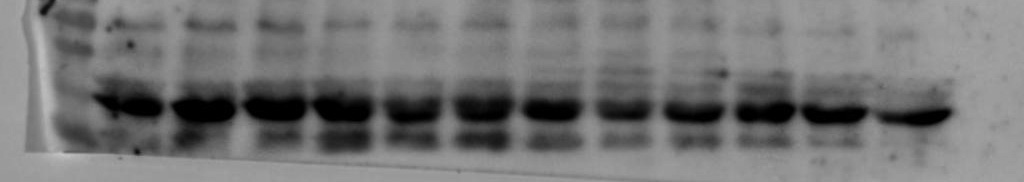

Supplement: S5 Fig — (JPG) [file pone.0214680.s006.jpg]

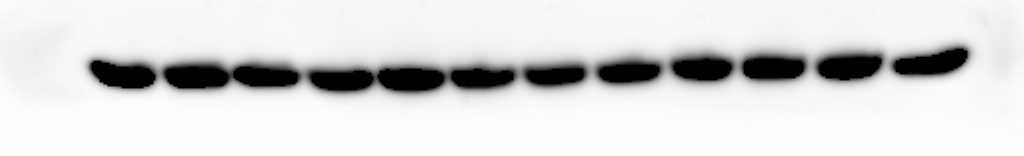

Supplement: S6 Fig — (TIF) [file pone.0214680.s007.tif]

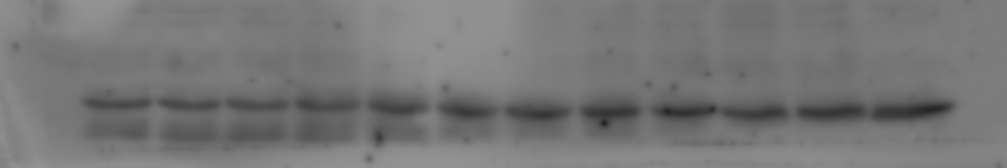

Supplement: S7 Fig — (TIF) [file pone.0214680.s008.tif]

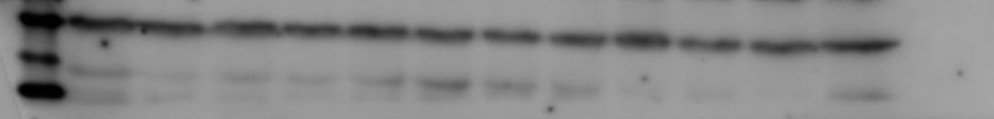

Supplement: S8 Fig — (JPG) [file pone.0214680.s009.jpg]

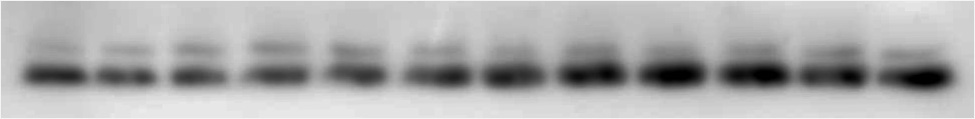

Supplement: S9 Fig — (JPG) [file pone.0214680.s010.jpg]

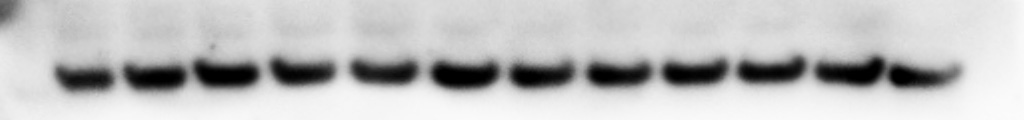

Supplement: S10 Fig — (JPG) [file pone.0214680.s011.jpg]
